# The Impact of Immunoglobulin G1 Fc Sialylation on Backbone Amide H/D Exchange

**DOI:** 10.3390/antib8040049

**Published:** 2019-10-01

**Authors:** Felix Kuhne, Lea Bonnington, Sebastian Malik, Marco Thomann, Cecile Avenal, Florian Cymer, Harald Wegele, Dietmar Reusch, Michael Mormann, Patrick Bulau

**Affiliations:** 1 Pharma Technical Development, Roche Diagnostics GmbH, Nonnenwald 2, 82377 Penzberg, Germany; felix.kuhne@roche.com (F.K.); lea.bonnington@roche.com (L.B.); sebastian.malik@roche.com (S.M.); marco.thomann@roche.com (M.T.); harald.wegele@roche.com (H.W.); dietmar.reusch@roche.com (D.R.); 2 Institute of Hygiene, University of Muenster, Robert-Koch-Strasse 41, 48149 Muenster, Germany; mmormann@uni-muenster.de; 3 Pharma Technical Development Analytics Biologics, F. Hoffmann-La Roche Ltd., 4070 Basel, Switzerland; cecile.avenal@roche.com (C.A.); florian.cymer@roche.com (F.C.)

**Keywords:** hydrogen/deuterium exchange, mass spectrometry, Fc glycosylation, antibody conformation, higher-order structure, biopharmaceutical, antibody effector function, FcγR binding, structure-function, sialic acid linkage

## Abstract

The usefulness of higher-order structural information provided by hydrogen/deuterium exchange-mass spectrometry (H/DX-MS) for the structural impact analyses of chemical and post-translational antibody modifications has been demonstrated in various studies. However, the structure–function assessment for protein drugs in biopharmaceutical research and development is often impeded by the relatively low-abundance (below 5%) of critical quality attributes or by overlapping effects of modifications, such as glycosylation, with chemical amino acid modifications; e.g., oxidation or deamidation. We present results demonstrating the applicability of the H/DX-MS technique to monitor conformational changes of specific Fc glycosylation variants produced by in vitro glyco-engineering technology. A trend towards less H/DX in Fc Cγ2 domain segments correlating with larger glycan structures could be confirmed. Furthermore, significant deuterium uptake differences and corresponding binding properties to Fc receptors (as monitored by SPR) between α-2,3- and α-2,6-sialylated Fc glycosylation variants were verified at sensitive levels.

## 1. Introduction

Glyco-engineering of the antibody fragment crystallizable (Fc) has improved the immune effector function of direct-targeting therapeutic antibodies (Ab) for almost a decade. The first glyco-engineered monoclonal antibody (mAb) was approved by the US Food and Drug Administration (FDA) in 2012. Mogamulizumab (Poteligeo^®^, Kyowa Hakko Kirin Co., Ltd., Tokyo, Japan) is a humanized anti-CC motif chemokine receptor 4 (CCR4) immunoglobulin G1 (IgG1) derived from Chinese hamster ovary (CHO) cells [1]. It was followed by Roche’s anti-CD20 obinutuzumab (Gazyva^®^) in 2013. Both drugs are predominantly afucosylated; i.e., the level of N-glycan core fucosylation (Fuc) is significantly reduced. An enhanced antibody-dependent cellular cytotoxicity (ADCC) of up to 100-fold compared to its fucosylated counterpart, was the rationale for this Fc engineering milestone [2]. This improved Fc effector function was proposed to be induced by closer carbohydrate contacts between the antibody Fc and the FcγRIII immune receptor glycans [3].

**Figure 1:**
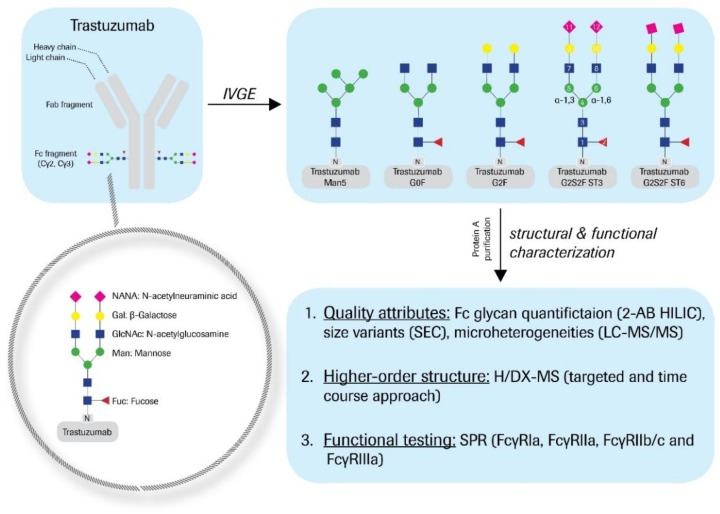
Study design. Trastuzumab drug substance (DS) was in vitro glyco-engineered (IVGE) with individual enzymatic post-process treatment, as described in the methods section. The trastuzumab Fc glyco-variants obtained (as determined by 2-AB HILIC; see Table 1 for details): G0F (81%), G2F (83%), G2S2F ST3 (60%), and G2S2F ST6 (43%). An additional trastuzumab Man5 variant (88%) was prepared by kifunensine treatment during the fermentation processing. All glyco-variants were protein A purified after IVGE treatment and further structurally and functionally characterized with 2-AB HILIC, SEC, LC-MS/MS (quality attributes), HDX-MS (higher-order structure), and SPR (functional testing).

The Fc carbohydrate moiety is covalently linked to N297 (EU numbering [4]) in the C’E-loop of the antibody heavy chain Cγ2 domain. Its core is built of a chitobiose; i.e., two N-acetylglucosamines (GlcNAc), and three branched α-mannosyl residues (Man), either α-1,3 or α-1,6-linked to the core Man. Predominantly, Fc N-glycans are bi-antennary and core-fucosylated at the GlcNAc1 (Figure 1). The antennae consist of various monosaccharides, such as Man, GlcNAc, galactose (Gal), and/or sialic acid; e.g., N-acetylneuraminic acid (NANA). The main Fc glycan variants to be found in bio-therapeutic antibodies are G0F, G1F, and G2F; however, many minor variants, such as G0, G1, G2, M5, hM3F, G2S1F, and G2S2F are also generated during the bioprocess. The diversity of N-glycan structures greatly contributes to the product heterogeneity. Thus, glycan levels are monitored during manufacturing as a process consistency parameter. Furthermore, as described for mogamulizumab, the Fc glycosylation can affect the product’s immune effector function, thus needs to be evaluated as a critical quality attribute (CQA).

Interestingly, upon receptor binding of Abs, the Fc carbohydrate moiety does not interact with the FcγR protein structure directly [5,6,7]. As a consequence, the concept of fine-tuning the immune system’s effector function by regulating the Fc-glycosylation has been discussed [8,9,10]; i.e., Fc binding is naturally modulated through interactions of the specific Fc N-glycan structures with themselves and with the antibody protein structure. Many of those interactions are suspected to stabilize the conformation of the receptor-interacting Cγ2 domain. The Cγ2 C’E-loop’s flexibility has been described to increase with shorter carbohydrate structures [11,12,13], which in turn leads to decreased Fc binding activities [9,11,14]. Moreover, the stepwise enzymatic, as well as in silico glycan truncation, revealed a twisting motion of the two Cγ2 domains relative to each other and a decrease in distance between them with shorter glycans [11,12,15,16,17]. Taken together, the Fc-glycosylation is proposed to modulate and pre-organize the receptor binding interface for optimal effector function [13].

Upon complete removal of the Fc N-glycan, binding to the clinically relevant immune receptors (i.e., FcγRI, FcγRIIa/b, and FcγRIIIa/b present on various leucocytes) is considerably reduced or even completely eliminated [18,19]. Accordingly, de-glycosylation has been shown to have a major impact on the structural integrity of the Cγ2 domain [17]. With attached sugars, non-covalent interactions with the protein surface, mainly hydrophobic residues, increase [11]. Such site-specific interactions with the antibody framework have been described for the α-1,6-arm GlcNAc and Gal residues [11,20,21]. GlcNAc has been observed to interact with F243, K246, and T260 [11,21]. Accordingly, mutations of F241 and F243 led to reduced FcγRIII binding of hyper-galactosylated IgG-Fc molecules [9,10]. For di-galactosylated IgGs as well, the backbone amides of L243, F244, and K247 (according to EU numbering [4]: L242, F243, and K246) have been interpreted as less solvent accessible due to the interactions’ involvement [17,21]. In close agreement, doubly galactosylated Cγ2 domains have been found to be arranged at a maximal distance to each other, which may be a favored interface conformation for FcγRIII interaction [11]. In any case, although less effective than afucosylation, hyper-galactosylation increases the mAb effector function [22,23]. Indeed, patients with rheumatoid arthritis have been reported to exhibit more IgGs with G1F and G2F compared to healthy patients [24].

Sialylation of the N-glycan termini has been demonstrated to regulate the immune modulatory feature (towards anti-inflammatory properties) of so-called intravenous immunoglobulins (IVIGs), a mixed fraction of healthy donor IgGs for the treatment of immunoglobulin deficiency and various immune-mediated diseases; e.g., chronic inflammatory demyelinating polyneuropathy (CIDP) [25,26,27,28]. Approximately 10%–11% of human IVIGs are sialylated and 1%–4% are di-sialylated [26,27,29]. The switch from pro to anti-inflammatory behavior has been ascribed again to conformational changes in the Cγ2 domains and has been supported by functional and structural data [30,31]. Several studies observed reduced binding affinities of sialylated IgGs for FcγRs and their consequent reduction in cytotoxicity [26,31,32], whereas other reports did not demonstrate functional changes upon terminal sialylation [23,33,34]. Independent from Fc function, an increase in inter-domain flexibility, including the reorientation of residue F241, and a slightly extended motion of the glycan have been described [6,30]. In a more recent in silico study, Harbison et al. demonstrated α-2,6-linkages to show a higher conformational freedom, which allows for the increased motion of terminal sialic acids α-2,6-linked to galactoses [35].

Many biophysical methods fail to detect such minor conformational changes of large bio-molecules, as they provide a global view on protein structure. The traditional analytical methods for monitoring higher-order structures (HOS), such as circular dichroism (CD), Fourier-transform infrared spectroscopy (FTIR), UV, and fluorescence spectroscopy, are insufficiently sensitive to detect minor spatial changes in large proteins such as antibodies, and rather, deliver information only pertaining to the entire averaged molecule. Changes induced in the HOS through chemical or post-translational modifications (PTM) may be limited to specific local regions in the protein, which, despite not affecting the overall structure, may still influence the bioactivity or pharmacokinetic (PK) behavior of a bio-therapeutic drug. Methods with an adequate structural resolution capability, e.g., X-ray crystallography, fail to provide dynamic information and are quite time-consuming. NMR facilitates the investigation of molecular dynamics, but is restricted in terms of molecule size and sample concentration [15]. The potential of hydrogen/deuterium exchange-mass spectrometry (H/DX-MS) as an alternative method to these traditional approaches has been demonstrated widely in the literature [36]. Hydrogen/deuterium exchange (H/DX) monitors protein backbone dynamics, as amide-bound hydrogens exchange according to hydrogen bond formation and/or solvent accessibility. HDX-MS, therefore, screens static and dynamic molecular alterations that influence the protein backbone.

Meanwhile, the application of H/DX-MS in the biopharmaceutical industry has become well-established. Many examples of H/DX-MS applied to structural changes in biopharmaceutical proteins [37,38], including mAbs [17,39,40,41,42] exposed to extreme stress conditions, have been published. The impact of chemical modifications and PTMs on the backbone amide hydrogen exchange behavior have been studied intensively [17,21,40,43]; e.g., for methionine oxidation [17,40,43], de-glycosylation [44,45], de-/hyper-galactosylation [17], high mannose variants [21,46], and afucosylation [17]. Likewise, the applications of H/DX-MS to characterizing mAb disulfide isoforms [47] and aggregates [48] have also been reported. The utilization of H/DX-MS for epitope mapping and ligand binding investigations of therapeutic proteins is especially well-established [49,50]. Recently, an improved H/DX-MS workflow for the detection of down to 1% Fc oxidation (M252) was published [43]. With the developed approach described, we now demonstrate the applicability of the H/DX-MS technique to monitor relevant structural changes using enzymatically altered Fc glycosylations, created by the previously introduced, in vitro glyco-engineering (IVGE) technology [23]. As a model system for the specific CQA assessment of bio-therapeutic proteins, the conformational impact of defined Fc glycosylations on antibody effector function was systematically examined in this study. Trastuzumab (Herceptin^®^) was chosen as an appropriate model for the investigation, due to the existence of extensive analytical and characterization data, in-house and in the literature.

## 2. Materials and Methods

### 2.1. Enzymatic Preparation of Fc Glycan Variants

Trastuzumab was expressed from Chinese hamster ovary (CHO) cells and formulated at 25 mg/mL in 60 mM histidine-HCl buffer, pH 6.0. Bioprocessing was conducted for Herceptin^®^ manufacturing. Post-process enzymatic treatment of trastuzumab (starting material) was performed as follows. The hypo-galactosylated “G0F” variant was generated by addition of 1.5 mL β-1,4-galactosidase (200 mU, ProZyme Inc., Hayward, CA, USA) to 190 mg starting material and subsequent incubation at 37 °C for 24 h. For the hyper-galactosylated “G2F” variant, 1.4 g starting material was mixed with 220 mL reaction buffer (10 mM UDP-Gal, 5 mM MnCl_2_, 100 mM MES, pH 6.5) and 42 mg β-1,4-galactosyltransferase (5.5 ± 0.5 mg/mL, Roche Diagnostics GmbH, Mannheim, Germany), and incubated at 32 °C for 28 h. Further, 205 mg CMP-NANA (c = 4 mg/mL in H_2_O) and 41 mg α-2,3-sialyltransferase or α-2,6-sialyltransferase (5.5 ± 0.5 mg/mL, Roche Diagnostics GmbH) were added to 410 mg of the G2F variant to obtain the “ST3” or the “ST6” variant, respectively. The samples were then diluted with 200 nM alkaline phosphatase (AP) and 0.1 mM ZnCl_2_ (final concentrations), and subsequently incubated at 37 °C for 24 h. For the ST6 variant, another 41 mg α-2,6-sialyltransferase and 200 mg CMP-NANA (c = 10 mg/mL in H_2_O), as well as AP and ZnCl_2_ (final concentrations: 264 nM and 0.1 mM) were added. The ST6 sample was then again incubated at 37 °C for 17 h. To obtain the trastuzumab “Man5” high mannose variant, 0.1 µg/mL kifunensine was added during cell cultivation (titer: 0.58 mg/mL trastuzumab). Purified material (mainly M5–M9 variants) was buffer-exchanged with 0.1 M sodium acetate, 0.5 mM CaCl_2_ (pH 5.0) and enzymatically glyco-engineered by the addition of 26 µg α-1,2-mannosidase (Genentech Inc., South San Francisco, CA, USA) per mg mAb (to obtain predominantly M5 variants). The reaction was performed at 37 °C for 24 h. The de-glycosylated variant (hereinafter denoted as “Degly”) was obtained by treatment of 380 mg starting material with 1.9 mL PNGaseF (250 U, Roche Diagnostics GmbH), further formulated with 10 mM sodium phosphate, pH 7.2, and incubated at 37 °C for 24 h. The starting material was also utilized as reference material “RM” throughout all experiments. Therefore, trastuzumab was incubated in drug substance (DS) sample buffer at 37 °C for 24 h and otherwise treated analogously. All samples were finally purified by Protein A chromatography and buffer-exchanged with DS sample buffer.

### 2.2. Analysis of Trastuzumab Quality Attributes

For the quantification of individual glycan species, 200 µg trastuzumab sample was buffer-exchanged with 10 mM ammonium formate buffer (pH 8.6) and incubated with 2 µL PNGaseF (500,000 units/mL, New England Biolabs GmbH, Frankfurt, Germany) at 45 °C for 1 h. Glycan 2-aminobenzamide (2-AB) labeling was performed at 65 °C for 2 h (Signal™ 2-AB labeling kit, ProZyme Inc.). Labeled glycans were hydrophilic interaction chromatography (HILIC)-separated (Waters, BEH Glycan 1.7 µm, 2.1 × 150 mm) and fluorescence-detected on a Waters ACQUITY UPLC system, as recently described [51].

Liquid chromatography-mass spectrometry (LC-MS/MS) peptide mapping and the quantification of relevant amino acid modifications were principally conducted as previously described [52,53]. In brief, all samples were denatured with 8 M Gua-HCl (pH 6.0) and reduced with 10 μL (c = 0.1 g/mL) dithiothreitol (DTT) at 50 °C for 1 h. Samples were buffer-exchanged (0.02 M His-HCl, pH 6.0) and further digested with 10 µL (c = 0.25 mg/mL) trypsin (Roche Diagnostics GmbH) at 37 °C for 18 h. Peptide separation (BEH C_18_ 1.7 μm, 2.1 × 150 mm) was performed on a Waters ACQUITY UPLC system. Online mass spectrometric detection was generated with a Waters Synapt G2 HDMS Q-ToF mass spectrometer. For relative quantification of modified peptides, GRAMS AI (Thermo Fisher Scientific Inc., Waltham, USA) was used.

Size exclusion chromatography (SEC) was performed with 20 µg protein sample on a Waters Alliance HPLC instrument with a BioSuite™ column (250 Å, 5 µm, 7.8 × 300 mm). With mobile phases of 200 mM KH_2_PO_4_ and 250 mM KCl (pH 7.0), and at a flow rate of 0.5 mL/min, variants were UV_280_ detected and peak-integrated for the quantification of molecular weight species.

### 2.3. FcγR Binding Study

An in-house study on the Fc binding characteristics, as evaluated by surface plasmon resonance (SPR) analysis, has been recently published in the journal Bioanalysis (Future Science Ltd., London, UK) [54]. It covers the data sets discussed in this manuscript, but with a focus on functionality. The data was re-assessed for direct comparison to the starting material for the experiments herein. The interactions between the trastuzumab glycan variants and immobilized immune receptors generated (FcγRIa, FcγRIIa H131, FcγRIIa R131, FcγRIIb/c, FcγRIIIa F158, and FcγRIIIa V158) were measured with a Biacore T200^™^ instrument (GE Healthcare Inc.); procedure described in detail by Thomann et al. [54].

### 2.4. Higher-Order Structure (HOS) Characterization by Hydrogen/Deuterium Exchange-Mass Spectrometry (H/DX-MS)

For structural characterization, the trastuzumab variants were adjusted to 6.6 mg/mL in 10 mM KH_2_PO_4_/K_2_HPO_4_ buffer, pH 7.0. H/DX was achieved by 1:20 dilution in 10 mM K_2_HPO_4_/KH_2_PO_4_/D_2_O buffer, pH 7.0, at room temperature. The exchange reaction was quenched by 1:2 dilution with ice-cold 100 mM K_2_HPO_4_/KH_2_PO_4_, 500 mM TCEP, and 4 M guanidine, pH 2.4. The samples were immediately shock frozen on dry ice and stored at −80 °C.

For the targeted H/DX approach, exchange reactions were quenched after 10 min of H/DX. Three varying combinations, with *n* = 6 replicates per (glyco-)variant, were prepared with significant time intervals in between. Non-deuterated reference samples of all glycan variants were prepared in triplicate. For the time course H/DX approach, reactions were quenched after 0.5 min, 1 min, 10 min, 30 min, 1 h, 3 h, and 48 h. Deuterated and non-deuterated samples were prepared in triplicate.

All samples (of both H/DX approaches) were measured on a Waters nanoAcquity UPLC M-Class system with H/DX technology connected to a Waters Synapt G2 HDMS Q-ToF mass spectrometer. Each sample was thawed immediately prior to measurement. Sample injection (55 pmol) was performed manually. The coupled 2D-LC setup operates with online-digestion at 15 °C; subsequent trapping was at 0 °C on a Waters Acquity UPLC BEH C_18_ Van guard pre-column (1.7 μm, 2.1 × 5.0 mm); and final separation was on a Waters BEH C_18_ analytical column (1.7 μm, 1 × 100 mm). For online-digestion, either an immobilized pepsin/type XIII (NovaBioAssays LLC, Woburn, MA, USA) or Poroszyme™ pepsin column (Thermo Fisher Scientific Inc., Waltham, MA, USA) was used. Back-exchange (i.e., deuterium loss) was determined as 49% ± 14% using the 48 h labeling values as approximation for 100% exchange. The percentage difference of theoretical and measured deuterium uptake per peptide, was averaged for the whole IgG sequence. The H/DX data was not corrected for this deuterium loss, as only the relative levels of deuterium incorporation between the samples have been compared.

Peptide identification was performed with Waters ProteinLynx Global Server™ 3.0.2. The data was processed and analyzed with Waters DynamX 3.0.0. Detected charge states were averaged for the individual peptides. The relative deuterium uptake (average D uptake) per peptide [Da] was calculated compared to that of the non-deuterated samples. Uptake differences between samples were calculated by subtraction of the corresponding average uptake values. The reduction in H/DX was calculated by normalization on de-glycosylated trastuzumab (showing maximum exchange in affected protein regions and used as a system suitability test for every H/DX-MS experiment).

## 3. Results

The Fc glycan variants were generated by applying post-process enzymatic engineering to trastuzumab starting material (Figure 1). As described recently, this IVGE approach was accomplished by the systematic and differential use of commercially available recombinant enzymes [23]. The Fc glycan distribution was monitored by 2-AB labeling of the liberated oligosaccharides. The results are summarized in Table 1.

In detail, a hypo-galactosylated variant “G0F” (81% G0F), a hyper-galactosylated variant “G2F” (83% G2F), and two differentially sialylated variants “ST3” (60% G2S2F) and “ST6” (43% G2S2F) with α-2,3- or α-2,6-linked sialic acids were produced. A variant with significant, high mannose levels “Man5” (88% Man5) was also generated through the application of the cytotoxin kifunensine during the fermentation processing, followed by α-1,2-mannosidase treatment. In addition, a de-glycosylated sample “Degly” was prepared by PNGaseF treatment. Equally treated, but unprocessed trastuzumab reference material was utilized as a control sample “RM.”

To assess structurally relevant chemical and post-translational amino acid modifications, potentially induced by the IVGE sample processing, all samples generated were further evaluated by LC-MS/MS peptide mapping (Table 2). The difference in light chain N30 deamidation was quantified at a maximum variation of 2.2% relative abundance. The heavy chain D98 isomerization was determined within 3.0% variance. The corresponding succinimide formation was found to vary within 1.8% relative abundance. More importantly, the oxidation level for the susceptible residue M252 in the conserved IgG1 region was determined to be equally and only moderately elevated up to a maximum of 2.6%, for all IVGE samples compared to the trastuzumab reference material. In summary, no significant alterations for chemical and post-translational modifications were observed for the IVGE samples. In addition, the potential formation of antibody fragments and aggregates was investigated by size exclusion chromatography (SEC). Likewise, no alterations in distribution of molecular weight species compared to the trastuzumab reference material could be detected (Table 3).

**Figure 2:**
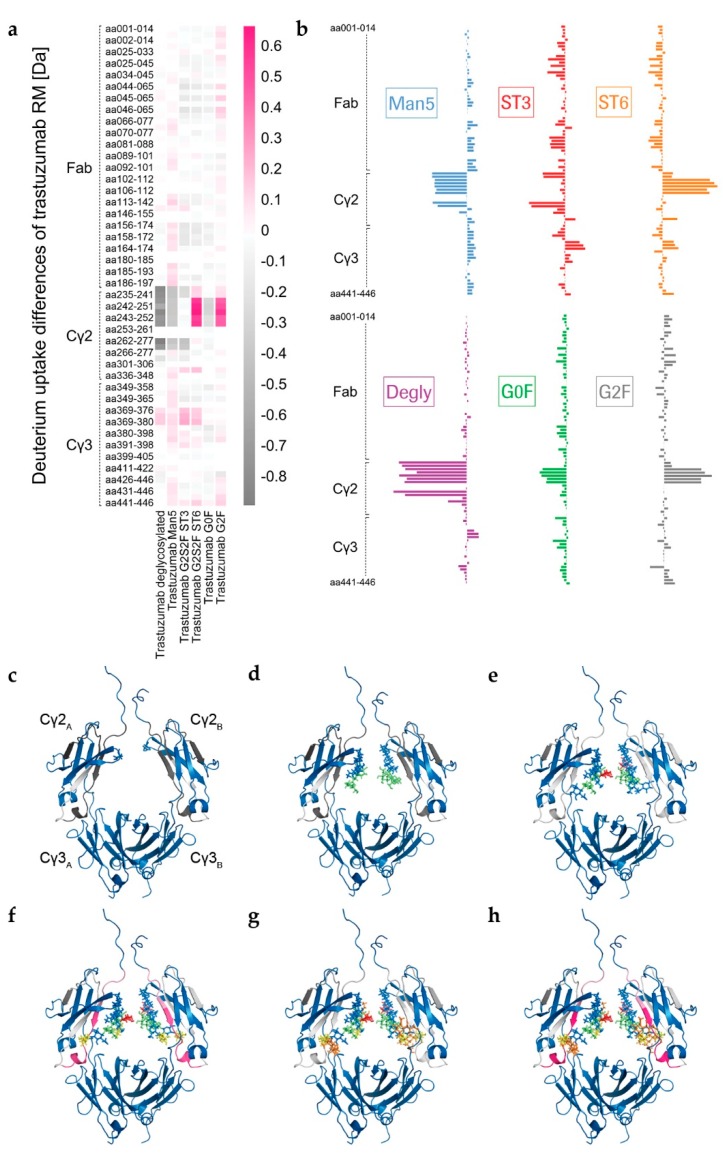
Average deuterium (D) uptake differences (Da) of trastuzumab reference material (RM) and glyco-engineered trastuzumab variants (*n* = 6) after 10 min of H/DX. (**a**) Differential heat map and (**b**) uptake plot of (shared) trastuzumab heavy chain peptides, resulting from pepsin/type XIII digestion (sequence coverage 87%–94%). (**c**–**h**) Differential D, uptake as established in (**a**) and projected onto Fc crystal structures based on PDB ID code 5VGP: (**c**) “Degly”, (**d**) “Man5”, (**e**) “G0F”, (**f**) “G2F”, (**g**) “ST3”, and (**h**) “ST6”.

**Figure 3:**
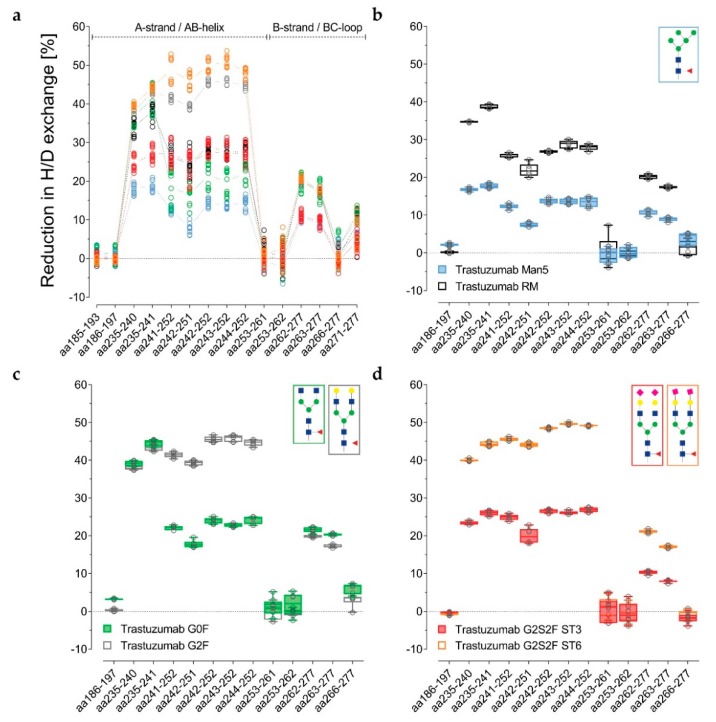
Reduction in H/DX (%) of trastuzumab glycan variant Cγ2 domain peptides, resulting from pepsin or pepsin/type XIII digestion (sequence coverage 87%–94%), normalized with the de-glycosylated trastuzumab sample. (**a**) Single peptide values of three targeted (10 min H/DX) experiments with trastuzumab Man5 (light blue), G0F (green), RM (black), ST3 (red), G2F (gray), and ST6 (orange). (**b**–**d**) Box plots with single peptide values of single experiments (*n* = 6) and representing boxes showing minimum, 25th percentile, median, 75th percentile, and maximum values. Additional statistical significance testing was as recently described by Hagemann et al.; see Figure S3 [60].

The Fc glycan variants were further analyzed by a systematic structural and functional characterization approach. An optimized targeted H/DX-MS approach for the detectability of minor structural changes within a set time range was established, as recently described [43]. An optimal deuterium (D) incubation time of 10 min was determined (data not shown). The 2D LC-MS/MS peptide mapping (online pepsin/type XIII protease digestion and subsequent RP C_18_ separation) yielded sequence coverages of 87%–94% and 81%–98% for the non-deuterated antibody heavy and light chains, respectively. For each glycan variant over all three data sets was collected, and generated by two independent operators and with two different digestion columns, corresponding to up to 18 replicates for each sample (Figure 2 and Figure 3).

The visualization of glycan-induced structural changes was realized by calculation of the relative deuterium uptake (D uptake) difference in [Da] for the RM sample (Figure 2). This representation is used here to facilitate identification of trends elicited by the various individual N-glycans compared to a “standard” heterogeneous mixture (RM). Most of the changes were seen for the heavy chain Cγ2 domain, but minor changes were for the heavy chain Cγ3 domain, and none were for the light chain (Figure 2 and Figure S1). The sialylated variants ST3 and ST6, as well as the Degly variant, all showed less H/DX within the Cγ3 domain region V369-E380 compared to the RM (Figure 2). The Cγ2 domain uptake differences were observed for the peptides with amino acids (aa) 235-240, aa235–241, aa241–252, aa242-251, aa242–252, aa243-252, aa244-252, aa262-277, and aa263-277 (for EU numbering [4] for shared peptic/type XIII peptides of three targeted experiments, see Table S1). Affected areas are the A-strand, the following AB-helix, the B-strand, the BC-loop and the C-strand of the Cγ2 domain (Figure 2c–h, structure based on PDB ID code: 5VGP). Three distinct regions with significant uptake differences can be described: L235–F241 (hinge/A-strand), F241–M252 (A-strand/AB-helix), and V262–W277 (B-strand/BC-loop/C-strand), graphically illustrated in Figure 3a. No differences could be detected for the region I253–V262, which contains the intra-domain disulfide bond between C261 and C321 [12]. Further, the glyco-peptide uptake differences were not determined between the different variants due to the diversity of glycan structures. The varying number of H/D exchanging acetamido groups on the individual glycans makes it impossible to quantitatively compare peptides within the C’E-loop area, i.e., the glycosylation site, as has been described in detail by Guttman et al. and More et al. [46,55].

**Figure 4:**
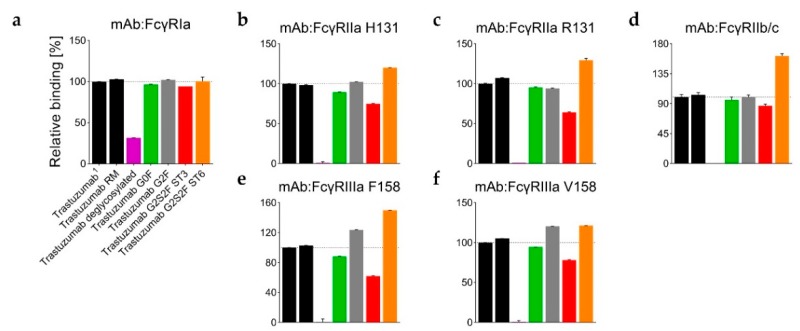
(**a**–**f**) Relative FcγR binding (%) of trastuzumab (glyco-)variants, as normalized with ^1^ trastuzumab starting material. Binding affinities were measured by surface plasmon resonance (SPR) technology; *n* = 3.

In absence of glycan structures, the Cγ2 domain has been repeatedly shown to be susceptible to H/DX [17,44,45,46]. Accordingly, Degly incorporated considerably more deuterium than RM (up to Δ−0.9 Da per peptide within the Cγ2 regions L235–M252 and V262–W277, see Table S1). The corresponding uptake differences per peptide were negative, as shown for the RM (Figure 2a–c). As demonstrated in literature before [17,19,56,57,58], the Fc binding activity was found to be almost completely lost in the absence of the Fc glycan (Figure 4 and Table S2). Only a weak relative binding activity for the FcγRIa receptor remained (Figure 4a). With an uptake difference of Δ−0.4 Da (L235–M252 and V262–W277), the RM also incorporated less deuterium than the Man5 sample (Figure 2a,b,d and Table S1). In contrast, hyper-galactosylation (G2F) and α-2,6-sialylation (ST6) stabilized and/or shielded the A-strand and the AB-helix; i.e., incorporated even less deuterium than the RM (Figure 2a,b,f,h). In the region F241–M252, the RM exhibited an average uptake difference of Δ0.5 Da, compared to the G2F variant, and Δ0.6 Da compared to the ST6 sample (Table S1). Interestingly, the Cγ2 region F241–M252 of the ST3 variant did not reveal uptake differences relative to the RM (Figure 2a,b,g). Uptake differences of Δ−0.3 and Δ−0.4 Da were calculated for the other two regions L235–F241 and V262–W277, respectively. Further, the trastuzumab RM exhibited an uptake difference of Δ−0.3 Da within the A-strand/AB-helix (aa241–252) compared with the G0F variant.

The data are presented in Figure 3a–d alternatively, normalized with the de-glycosylated trastuzumab sample. The reduction in H/DX was calculated, as correlated with the successive structural extension of the Fc glycan. Several peptides for the three distinct regions are shown. For the RM, the H/DX of the representative peptide aa235–241 was reduced by 38% ± 2% (Figure 3a and Table S1). The representative peptides aa241–252 and aa262–277 showed 26% ± 2% and 20% ± 1% reductions, respectively. With an 18% ± 4% reduction in H/DX, the hypo-galactosylated G0F variant was found to exhibit slightly lower values for the aa241–252 peptide (Figure 3a and Table S1). Accordingly, the G0F sample revealed a similar relative binding activity for FcγRI-III receptors compared to the RM (Figure 4b–f). For the Man5 sample, a significantly lower H/DX reduction of 13% ± 1% for the aa241–252 peptide was observed. An equivalent offset could be observed for the Man5 Cγ2 BC-loop area V262–W277, with an 11% ± 1% (versus 20% ± 1% for the RM) reduction in H/DX, and an even more significant shift for the hinge/A-strand segment, including residues L235–F241 with a 19% ± 1% (versus 38% ± 2% for the RM) reduction in H/DX (Figure 3b).

As stated earlier, the hyper-galactosylated G2F variant was found to be less prone to H/DX within its Cγ2 domain (41% ± 1% versus 26 ± 2% (RM) reduction in H/DX for aa241–252). This is in contrast to the hypo-galactosylated G0F and high mannose Man5 variants. The presence of galactoses appears to reduce the exchange of amide-bound hydrogens within the Cγ2 A-strand/AB-helix region (Figure 3a,c). The sialylated ST6 variant exhibits even further diminished Cγ2 dynamics/accessibility (49% ± 3% reduction in H/DX for the aa241–252 peptide; Figure 3a,d). Correspondingly, the G2F and ST6 variants also revealed different relative binding activities for FcγR receptors (Figure 4). In detail, the binding affinity to the FcγRIIIa (V158 and F158) was found to be enhanced for the G2F variant (120% ± 1% and 124% ± 1%; see Table S2). In addition, the ST6 variant showed increased binding activity for the FcγRIIa R131 (130% ± 2%), FcγRIIa H131 (120% ± 1%), FcγRIIb/c (162% ± 4%), FcγRIIIa V158 (121% ± 1%), and the FcγRIIIa F158 (150% ± 1%) compared to the trastuzumab starting material (Table S2, Figure 4b–f).

Interestingly, the α-2,3-linked G2S2F variant (ST3) revealed an opposite trend for H/DX and FcγR receptor binding activity compared to the ST6 variant. The regions L235–F241 and V262–W277 showed 27% ± 2% (versus 38 ± 2% for the RM) and 11% ± 1% (versus 20 ± 1% for the RM) reductions in H/DX. With a 27% ± 3% reduction in H/DX, the area including the A-strand and flanking the AB-helix (F241–M252) exhibited a similar exchange behavior as observed for the RM (Figure 3a). Accordingly, the ST3 sample revealed reduced binding affinity (up to around 40%) to the tested FcγIIa/b/c and FcγRIIIa receptors (Figure 4b–f), but had no significant effect on FcγRIa binding (Figure 4a). Most severely, the FcγRIIIa F158 interaction was found to be impaired, with 62% ± 1% relative binding (Figure 4e).

**Figure 5:**
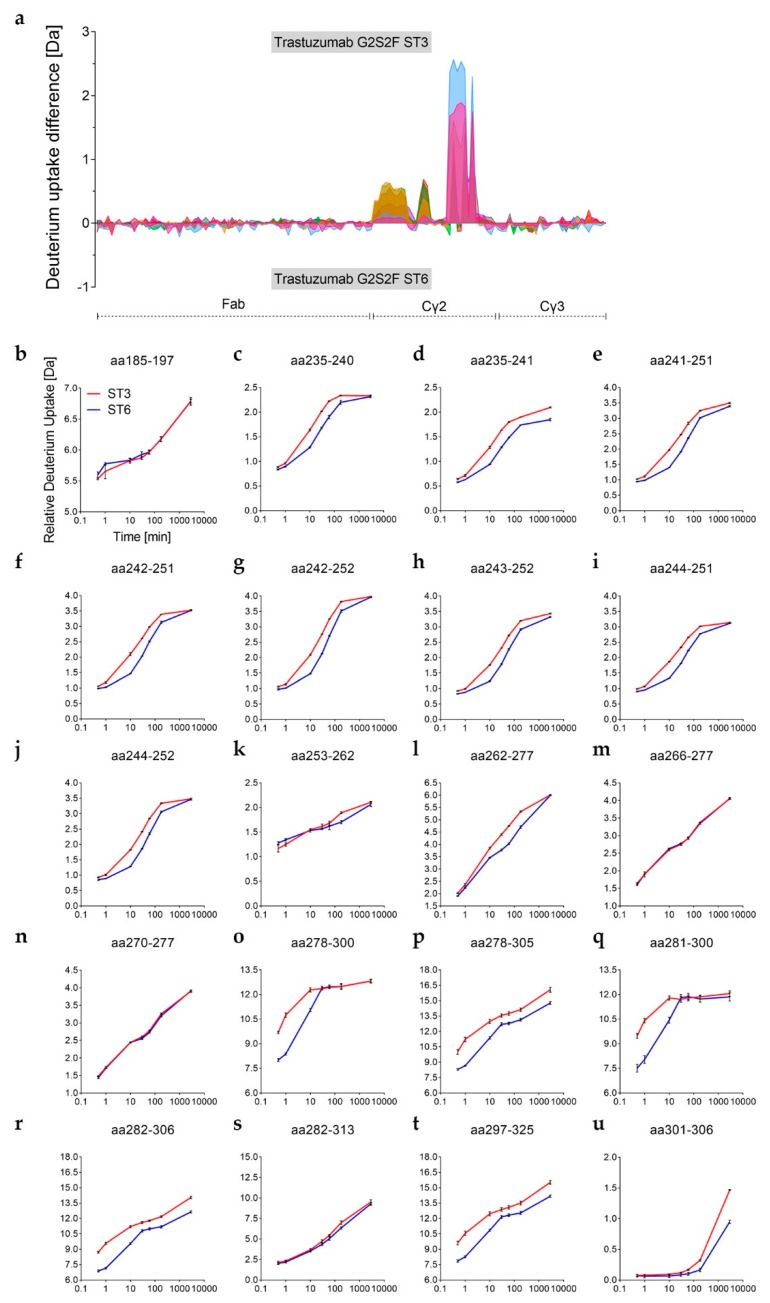
D uptake (Da) of differentially sialylated trastuzumab glycan variants (*n* = 3). Shown are heavy chain peptides, as obtained by pepsin/type XIII digestion (sequence coverage 88%). (**a**) Uptake difference plot of H/DX time course experiment performed for 0.5 min (pink), 1 min (light blue), 10 min (orange), 30 min (green), 1 h (red), 3 h (purple), and 48 h (black). (**b**–**u**) Relative uptake curves of individual peptides, as summarized in (**a**).

We further analyzed the observed differences in H/DX and corresponding binding affinities for Fcγ receptors between the ST3 and ST6 variants by an expanded H/DX approach (time course experiment). The differences in H/DX between the α-2,3 and the α-2,6-sialylated trastuzumab variants observed at 10 min deuterium incubation time (Figure 3d) could be verified for the additional time points investigated (0.5 min, 1 min, 10 min, 30 min, 1 h, 3 h, and 48 h), as shown in Figure 5. Sequence coverages of 88% for both the heavy and the light chains were achieved. As illustrated in Figure 5, the two G2S2F variants revealed significant uptake differences for the heavy chain Cγ2 domain regions L235–M252, V262–W277, and Y278-N325 (no changes were seen for the light chain as shown in Figure S2). The respective values and peptic/type XIII peptides are listed in the Supplementary Table S3. The three regions of varying D uptake also revealed differences in the exchange rates (Figure 5a). The corresponding relative D uptake curves are depicted in Figure 5b–u. For the residues L235–M252, the maximum D uptake difference between the two sialic acid variants was already reached after 10 min (Figure 5c–j). With an uptake difference of Δ0.3 Da for the aa235–241 and Δ0.6 Da for the aa242–252 peptides, the ST3 variant exchanged more hydrogen than the α-2,6-linked ST6 variant in this region (Figure 5d,g and Table S3). Notably, only the peptide aa235–241 exhibited further H/D exchange beyond 60 min in this region (Figure 5d). The area V262–W277 (e.g., aa263–275) followed an overall faster exchange rate, but the uptake difference maximum appeared later (after 60 min with maximum Δ0.7 Da uptake difference; see Figure 5l and Table S3). This explains the overall lower H/DX differences observed in the preceding 10 min targeted approach for that sequence area (V262–W277), as shown in Figure 3a. The uptake difference maximum for the glycosylated region Y278–N325 (C-strand to FG-loop) was reached even more rapidly (Figure 5a). Comparison of the doubly sialylated glyco-peptide revealed more D uptake within the C’E-loop (E294-R301) for several ST3 variant peptides within the region (Figure 5o–t). In detail, maximum uptake differences compared to the ST6 variant were observed already after 1 min; e.g., with Δ2.6 Da for the peptide aa278–305 (Figure 5p). We further found that amino acid regions adjacent to the glycosylation site showed slow H/D exchange rates at incubation times <60 min. As visible for the heavy chain peptide aa301–306 (Figure 5u), a significant exchange difference can only be detected after 3 h (Δ0.2 Da) and 48 h (Δ0.5 Da). Together, this indicates that the α-2,3-linkage impacts the glycan dynamics more significantly, resulting in an increased motion of the Cγ2 C’E-loop area compared to the α-2,6-linkage.

Overall, the time course data identifies the 10 min deuterium incubation time, selected for the targeted H/DX experiments, as suitable for the detection of H/DX differences caused by different N-glycosylations. Moreover, the structural observations for ST3 versus ST6 are in close agreement with the differential binding activities for FcγR receptors, as probed by SPR (Figure 4).

## 4. Discussion

Many studies correlating structural observations with the effector function of IgGs have been published [61]. The idea of passive functional regulation through the Fc-glycan structure has already been shaped [8,11,13,17]. The present study aims at the systematic comparison of common N-glycan variants. By means of in vitro glyco-engineering (IVGE), highly comparable samples, varying only through their defined glycan structure, were generated (Figure 1) [23]. Compared to trastuzumab starting material, no substantial chemical modifications or structural degradations of the primary structure were found to be induced by the enzymatic treatment (Table 2 and Table 3).

We recently presented a targeted H/DX-MS approach to monitor minor structural alterations, wherein reliable assignments of H/DX differences resulting from methionine oxidation were demonstrated. M252-ox levels to <5% could be differentiated, and as such, the impact of methionine oxidation cannot be excluded here entirely [43]. However, no correlation between the oxidation levels and the D uptake trends observed here could be established. The H/DX-MS approach described was utilized here to compare overall seven glyco-variants. After 10 min of deuterium incubation, significant uptake differences could be attributed to the individual glycan structures (Figure 2). We merged three internally normalized data sets for direct comparison and were able to demonstrate high method robustness (Figure 3).

Whereas no exchange differences could be established for the antibody light chain (Figure S1) or Fab part of the heavy chain, five regions exhibiting differential uptake were identified in the heavy chain Fc part (including the C’E loop area, which could only be analyzed for equal glycosylations). Minor changes could be determined for the Cγ3 domain, with the BC-loop region VKGFYPSDIAVE (aa369–380) of the de-glycosylated and both sialylated variants incorporating slightly less deuterium than the RM (Figure 2a). Differential H/DX in this region has not previously been described in the literature. However, the main uptake differences observed here were within the Cγ2 domain, which is consistent with previous reports investigating antibody glyco-variants [17,21,44,45,46]. The Cγ2 domain regions revealing differential uptake with our glyco-variant panel correspond to the A-strand, AB-helix, B-strand, and BC-loop (Figure 2c–h and Figure 3a). Specifically, the domain regions LGGPSVF (aa235–241), FLFPPKPKDTLM (aa241–252) and VVVDVSHEDPEVKFNW (aa262–277) showed differential exchange, confirmed by overlapping peptides (Figure 3a). Since the peptide aa266–277 exhibited no significant exchange differences, the differences observed for the peptide aa262–277 can be attributed to its VVVD segment (V262–D265) by subtraction.

H/DX differences upon de-glycosylation have been extensively investigated, with the region L235–M252 described [44,45]. Subsequent loss of the antibody effector function has also been observed for fully glycan truncated Fc [17,19,56,57,58]. Our data confirms this, with de-glycosylated trastuzumab incorporating more deuterium in the regions L235–F241, F241–M252 and V262–D265 (Figure 2) and showing loss of effector function (Figure 4).

Fang et al. reported increased D uptake for IgG2 high mannose variants compared with heterogeneous glycan samples (~60% G0F, ~30% G1F, and ~4% G2F). They identified the peptides F241–M252 and V263–F275, which correspond to our peptides FLFPPKPKDTLM (aa241–252) and VVDVSHEDPEVKFNW (aa263–277), shown in Figure 3a [21]. The results from More et al. augmented these findings and also showed uptake differences for the hinge-Cγ2 interface and Cγ3 domain peptides, in their comparison of IgG1 de-glycosylated, GlcNAc, and Man5 variants with high mannose (Man5-Man9) material [46]. We generated a sample with ~90% Fc Man5 and demonstrated increased deuterium incorporation compared to the isolated G0F, G2F, and G2S2F variants, and the heterogeneous RM fraction in the areas described by Fang and colleagues (Figure 3a–b) [21]. Furthermore, we demonstrate an increased D uptake for the peptide LGGPSVF (aa235–241), an area described to have differential uptake behavior by More et al. 2018 [46]. A functional correlation with fucosylated high mannose mAb has not been reported in the literature and was also not addressed in our study. The previously reported increase in FcγRIII binding and in vitro ADCC has only been demonstrated for afucosylated high mannose variants [62,63,64]. Our data suggests no relevant fucose-initiated interactions to be determining for the Fc dynamics, thus pre-defining an optimal binding interface for FcγR interaction. This supports the concept of steric hindrance by the core fucose during FcγRIIIa interaction [3]. Moreover, an increase in serum clearance of high mannose variants has been reported [65]. HD/X interaction studies with mannose receptors, FcRn and FcγR, could provide further clarification on this topic.

The H/D exchange behavior of our enriched G0F sample (~80% G0F) was quite comparable to trastuzumab RM (~40% G0F, ~40% G1F, and ~10% G2F) although it did show slightly more D uptake; i.e., less reduction in H/DX (Figure 3a). This appears to be reasonable in light of the decreased exchange observed for the trastuzumab G2F variant (Figure 3c). G2F (~85% G2F) with two additional galactose units, showed significantly less deuterium incorporation for the region FLFPPKPKDTLM (aa241–252), plus significantly enhanced FcγRIIIa binding (Figure 4e–f). These findings are consistent with previous studies. Houde et al. reported the same peptide to exchange more hydrogen upon galactosylation [17]. Due to peptic peptide overlaps, they were able to assign the increased H/DX to the backbone amides of the residues L243, F244, and K247 (according to EU numbering [4]: L242, F243, and K246). Further they reported a strong increase in relative binding to FcγRIIIa upon hyper-galactosylation. Krapp et al. also describe a stable GlcNAc–F241 interaction [11]. Our data conforms both of those proposed interactions, with an uptake difference between G0F and G2F for aa241–252 but not for aa235–241 (Figure 3c). Subedi et al. further reported that F241/F243 mutations were impaired FcγR binding exclusively in the presence of terminal galactoses [9]. A potential explanation is that α-1,6-arm Gal preferentially interacts with F241/243. The glycan α-1,6-arm is described to be critical for protein surface contacts and has been simulated to adopt a “foldover” conformation upon galactosylation, whilst the α-1,3-arm extends between the Cγ2 domains in an “outstretched” and more rigid fashion [8,20,35].

We further present two highly sialylated but distinct trastuzumab glyco-variants comprised of ~60% α-2,3-linked G2S2F (ST3) or ~40% α-2,6-linked G2S2F (ST6), as the main component (Table 1). The ST6 variant showed relatively comparable H/DX behavior to the G2F variant, revealing, however, the overall least exchange of all glycan variants investigated (Figure 3a). In parallel, the ST6 variant exhibited the highest binding activity in most of the functional assays (Figure 4b–e). No effect of terminal sialic acids on the binding affinity to Fcγ receptors has been reported [23,26,33,34]. Further, the association of overall decreasing Cγ2 stability with decreasing glycan size [11,15,46] is in contrast to results presented by Ahmed et al. 2014 for the α-2,6 variant investigated [30]. The comparative results of the ST6 variant in our results indicate a positive correlation between stability of the Cγ2 region, as reflected by reduced H/DX, and enhanced functional activity. The differential exchange for the aa241–252 peptide between G2F and ST6 (Figure 3a) further supports the concept of dynamic modulation by differential glycan–protein and glycan–glycan interactions. In addition, an enhanced ADCC activity for the α-2,6-sialylated G2S2F variant has been reported the most recently [54]. The finding of decreased H/DX for doubly α-2,6-sialylated Fc glycan structures has not previously been described in literature and potentially throws a new light on the subject matter of anti-inflammatory IVIGs [66]. Our results indicate that the Cγ2 (and Cγ3) dynamics are not the only determining factor for specific and functional-defining interactions, as proposed for anti and pro-inflammatory effector functions, and consistent with previous work associating the lack of fucose with subsequently closer carbohydrate contacts [3,67]. Potentially, the sialic acid itself plays a crucial role in anti-inflammatory receptor binding, as observed for the SIGN-R1 [28].

For the α-2,3-linked sialic acid variant, D uptake differences could be observed for all of the aforementioned Cγ2 domain areas L235–F241, F241–M252, and V262–W277 (Figure 5c–m). While the peptide FLFPPKPKDTLM (aa241–252) showed an H/DX comparable to the mixed fraction RM, the peptides LGGPSVF (aa235–241) and the VVVD (aa262–265) segment showed more exchange compared to the RM (Figure 3a). The kind of linkage, therefore, can be shown to affect the H/DX kinetics. This trend was able to be further confirmed within an H/DX time survey (Figure 5a). The described areas exchanged relatively slowly (maximum H/DX difference about 10–30 min). Faster backbone hydrogen exchange was observed for the additional glyco-peptide region Y278–N325 (maximum H/DX difference ~1 min); e.g., for the peptide VEVHNAKTKPREEQY**N**STYRVVSVL (aa282–306) shown in Figure 5r. Very likely, the uptake is favored by the seven accessible acetamido groups of the G2S2F structure. However, the differential uptake of the glycan itself already reveals information about the glycan–glycan and glycan–protein interactions. We propose the α-2,6-structure is more shielded, due to (H-bonding) interactions (less H/DX), compared to the α-2,3-structure (more H/DX). The H/DX of the amide backbone of the C’E-loop peptides and the attached glycan structures cannot be differentiated. However, the large D uptake difference for the overall peptide does indicate an increased motion of the C’E-loop area for the α-2,3-linked G2S2F variant, which is proposed to determine the FcγR binding interface [5,11,13]. This would explain differential receptor interactions described for differentially linked sialic acids; e.g., for the CD22 binding [68]. Moreover, Anthony et al. recognized that IgG Fc α-2,3-linked sialic acid structures did not exhibit an anti-inflammatory potential [28,66]. This also correlates well with observations made by Falck and colleagues who report α-2,3-linked G2S1F IgG to be significantly more susceptible to tryptic digestion than α-2,6-linked G2S1F IVIGs [69]. The levels of the corresponding singly sialylated molecules in our samples were ~14% G2S1F (ST3) and ~27% G2S1F (ST6), respectively. In accordance with reports on differential sialylation of the bi-antennary glycan arms [70], we obtained about twice as much singly sialylated molecules for the α-2,6-linked variant compared to the α-2,3-linked version. A possible explanation could be an increased accessibility for the α-2,3-sialyltransferase due to decreased interaction of the ST3 glycan structure (predominantly the α-1,6-arm) with the protein surface. Indeed, our H/DX results support this assumption. The differing ratio of G2S1F/G2S2F for the two differentially linked sialic acid samples should also be considered in context of the observed results. With less conjugated sialic acids, more galactoses would be accessible to interact with the protein surface. In a recently published in silico study, Harbison et al. were able to show increased conformational freedom for α-2,6-linkages, as shown for an isolated G2S2F glycan structure [35]. Our results suggest (steric) hindrance of galactose interactions with the protein surface by the comparably short O-glycosidic linkage of the galactose C_3_ hydroxy-group and the sialic acid, e.g., the Gal interaction with K246 as presented by Houde et al. 2010. In contrast, the O-glycosidic linkage between the C_6_ hydroxymethyl-group (CH_2_OH) and the sialic acid protrudes out of the cyclic hexose conformation, which would yield a higher conformational degree of freedom [35].

## 5. Conclusions

Here, we present a combined approach of in vitro glyco-engineering and H/DX-MS to assess the impact of Fc glycosylation on higher-order structure (HOS). We were able to confirm significantly distinguishable uptake trends towards lower exchange rates for larger glycan structures. As an exception we found the G2S2F structure with terminal α-2,3-linked sialic acids to incorporate more deuterium compared to the α-2,6-linked sialic acid structure. A strong structure to function correlation between the H/DX and SPR data could be verified and highlights the important role of the terminal sialic acid linkage. As indicated by our results and as already suggested in other studies, the antibody backbone dynamics are most likely stabilized due to inter-glycan and glycan–protein interactions. Proposed is an overall entropic modulation towards an optimal binding interface. Described factors are the Cγ2 domain orientation and site-specific interactions, whereof the former is suggested to depend on the inter-glycan interactions and the latter on glycan–protein interactions. A key role has been ascribed to the fluctuation of the C’E-loop. Its motion supposedly depends on the Cγ2 dynamics and is again determined by certain secondary structure events.

Our results support this assumption. With more interactions favored by the individual structure, the overall backbone motion/accessibility decreases. The interactions facilitated by sialic acids and the mechanism for domain destabilization caused by the α-2,3-linkage of the terminal sugar should be further experimentally addressed. Our results show there is no strict correlation between Fc glycan size and resulting Cγ2 flexibility, with linkage, and hence, steric factors also contributing.

## Figures and Tables

**Table 1:** Relative quantification of 2-AB labeled (trastuzumab) N-glycans (2-AB HILIC).

Fc Glycosylation (%)	Trastuzumab ^1^	RM	Man5	G0F	G2F	G2S2F ST3	G2S2F ST6
G0	3.5	3.6	0.5	5.2	0.1	n.q.	0.4
G0F	34.2	39.3	3.4	80.6	n.q.	0.0	0.2
G1	2.8	2.9	1.0	0.1	0.1	0.2	0.7
G1F	41.9	39.0	3.3	1.4	0.4	0.1	0.1
G2	0.4	0.3	0.1	n.q.	5.4	0.5	0.4
G2F	9.3	7.2	0.8	0.7	83.1	1.6	1.8
G2S1	0.1	0.1	n.q.	0.2	n.q.	1.7	3.4
G2S1F	0.9	0.6	0.2	0.2	1.7	13.8	26.5
G2S2	0.1	n.q.	n.q.	n.q.	0.5	3.9	2.8
G2S2F	0.2	0.3	n.q.	0.1	n.q.	60.3	42.9
M3	n.q.	n.q.	0.1	n.q.	n.q.	n.q.	n.q.
M3F	n.q.	n.q.	0.1	n.q.	n.q.	n.q.	n.q.
hM3	0.4	0.6	0.2	1.0	0.0	0.1	0.3
hM3F	0.6	1.2	0.3	2.2	0.0	n.q.	0.2
hM3G1S1	0.1	0.1	n.q.	0.1	0.1	1.9	2.5
hM3G1S1F	0.3	0.4	n.q.	0.6	0.5	4.4	3.7
M4	n.q.	n.q.	0.1	n.q.	n.q.	n.q.	n.q.
hM4	0.3	0.3	n.q.	n.q.	1.0	0.1	1.2
hM4F	0.0	n.q.	n.q.	0.0	2.2	n.q.	n.q.
M5	1.3	1.3	88.3	1.4	1.4	3.1	3.3
hM5	n.q.	n.q.	n.q.	n.q.	n.q.	n.q.	n.q.
M6	n.q.	n.q.	n.q.	n.q.	n.q.	n.q.	n.q.
M7	n.q.	n.q.	n.q.	n.q.	n.q.	n.q.	n.q.
not assigned	3.9	2.8	1.4	6.3	3.5	8.3	9.7

^1^ Method reference standard; n.q. = not quantifiable.

**Table 2:** Relative quantification of chemical amino acid modifications (LC-MS peptide mapping).

Chemical Mod. (%)	Trastuzumab ^1^	RM	Degly	Man5	G0F	G2F	G2S2F ST3	G2S2F ST6
LC N30 ^2^ deamidation	9.6	8.7	10.9	9.1	9.1	9.0	9.6	10.0
LC N30 ^2^ succinimide	0.6	0.7	0.7	0.6	0.8	0.7	1.1	1.1
HC N54 ^2^ deamidation	1.5	1.6	1.7	2.2	1.7	2.0	1.8	1.6
HC N54 ^2^ succinimide	3.9	4.0	3.9	4.1	3.9	3.9	3.7	3.8
HC D98 ^2^ isomerization	7.7	7.6	8.0	10.0	7.0	7.7	7.9	8.7
HC D98 ^2^ succinimide	3.5	4.1	3.6	2.5	4.3	3.8	4.3	4.3
HC N389/390 ^3^ deam.	2.0	1.9	2.2	1.6	1.9	1.9	2.1	2.0
HC N389/390 ^3^ succ.	1.7	1.7	1.7	2.0	1.8	1.7	1.8	1.8
HC M252 ^3^ oxidation	2.3	2.6	3.4	5.2	3.0	3.8	3.8	3.8

^1^ Method reference standard; ^2^ Kabat numbering [59]; ^3^ EU numbering [4].

**Table 3:** Relative quantification of trastuzumab size variants (SEC-UV).

Mol. Weight Species (%)	Trastuzumab ^1^	RM	Degly	Man5	G0F	G2F	G2S2F ST3	G2S2F ST6
Monomer	99.8	99.6	99.6	99.4	99.6	99.3	99.2	99.1
total HMW ^2^	0.2	0.4	0.4	0.5	0.3	0.7	0.8	0.9
total LMW ^3^	0.0	0.0	0.1	0.1	0.1	0.0	0.0	0.1

^1^ Method reference standard; ^2^ high molecular weight species; ^3^ low molecular weight species.

## Supplementary Materials

The following are available online at https://www.mdpi.com/2073-4468/8/4/49/s1, Figure S1: Reduction in H/DX (%) of trastuzumab glycan variant peptides (whole sequence)*;* Figure S2: Uptake difference plot with D uptake (Da) of alternatively linked trastuzumab sialylation variants (light chain only); Figure S3: Volcano plot showing statistical significance testing of trastuzumab ST3 and ST6 D uptake differences; Table S1: D uptake differences and reduction in H/DX values; Table S2: Relative FcγR binding of trastuzumab glycan variants; Table S3: D uptake differences of trastuzumab G2S2F ST3 versus ST6.
