# Supplementary material for: The Impact of Immunoglobulin G1 Fc Sialylation on Backbone Amide H/D Exchange
Source: Antibodies (Basel). 2019 Oct 1;8(4):49. doi: 10.3390/antib8040049 (PMC6963987; doi:10.3390/antib8040049)
Supplement: Supplementary file 1 [file antibodies-08-00049-s001.pdf]

antibodies

MDPI

Article

Impact of immunoglobulin G1 Fc sialylation on backbone amide H/D exchange

Supplementary Materials

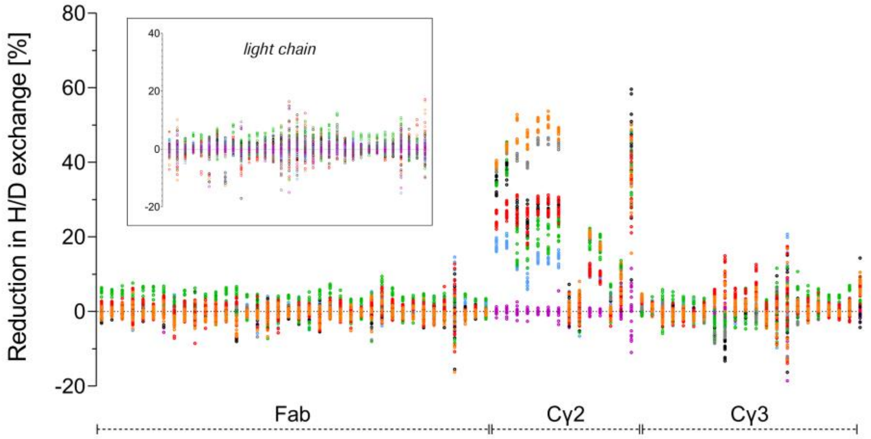

Figure S1. Reduction in H/DX [%] of trastuzumab glycan variant peptides *(whole sequence)*, resulting from pepsin and pepsin/type XIII digestion (sequence coverage 81-98% and 87-94% for light and heavy chains, respectively). The relative reduction of the different glycan variants was normalized with the de-glycosylated trastuzumab sample (Degly) data. Depicted are single peptide values (summarized in Table S1) of three targeted (10 min H/DX) experiments with trastuzumab Man5 (light blue), G0F (green), RM (black), ST3 (red), G2F (gray) and ST6 (orange) and Degly (purple). Only minor alterations in H/DX were observed for the analyzed light chain peptides (see inset).

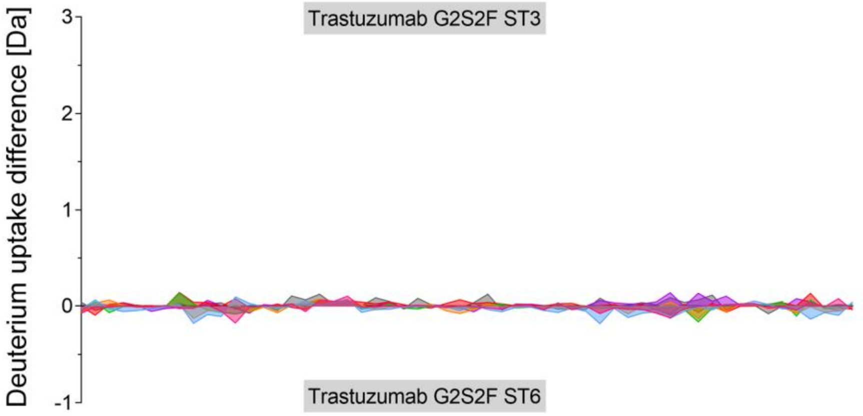

Figure S2. Uptake difference plot showing D uptake [Da] of alternatively linked trastuzumab sialylation variants *(light chain only)*. Shown are peptides (n=3) obtained by pepsin/type XIII digestion (sequence coverage 83%). The H/DX time course experiment was performed for 0.5 min (pink), 1 min (light blue), 10 min (orange), 30 min (green), 1 h (red), 3 h (purple) and 48 h (black).

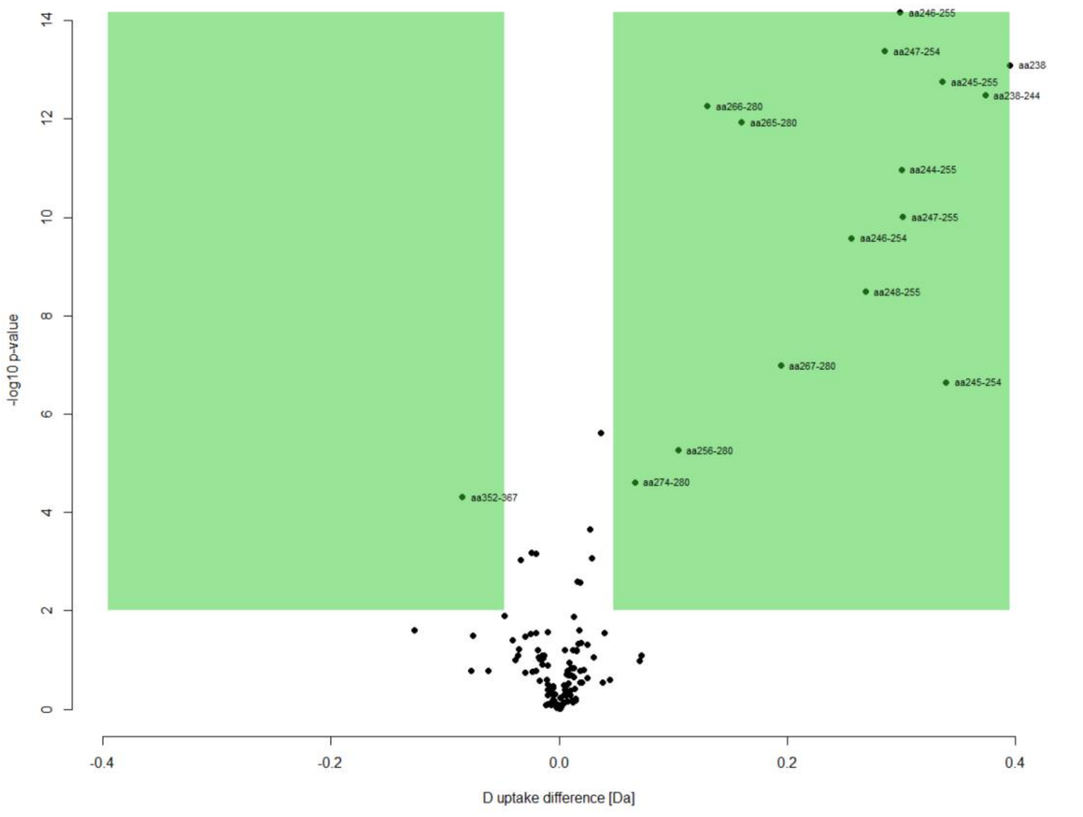

Figure S3. Volcano plot showing statistical significance testing of trastuzumab ST3 and ST6 D uptake differences, as proposed by Hagemann et al. [59]. For statistical significance testing of D uptake differences, Welch's *t*-test was performed (n=6). Corresponding *p*-values are depicted. Significant hits are located within the green rectangle and defined by an α=0.01 and a global significance threshold of ±0.05 Da, as calculated according to Hagemann et al. [59].

Table S1. D uptake differences and reduction in H/DX values, as shown in *Figure 2* and *Figure 3*.

	Trastuzumab Degly$^{(+++)}$	Trastuzumab RM$^{(+++)}$	Trastuzumab Man5$^{(++)}$	Trastuzumab ST3$^{(+++)}$	Trastuzumab ST6$^{(++)}$	Trastuzumab G0F$^{(++)}$	Trastuzumab G2F$^{(+)}$
	Deuterium uptake difference of trastuzumab RM [Da], average values of n=6 replicates						
aa186-197	0.0	-	0.1	0.0	0.0	0.0	0.0
aa235-240	-0.8	-	-0.4	-0.3	0.1	0.0	0.1
aa235-241	-0.8	-	-0.4	-0.3	0.1	0.0	0.1
aa241-252	-0.8	-	-0.4	0.0	0.6	-0.3	0.5
aa242-251	-0.6	-	-0.4	0.0	0.6	-0.3	0.5
aa242-252	-0.8	-	-0.4	0.0	0.7	-0.3	0.6
aa243-252	-0.7	-	-0.4	-0.1	0.5	-0.2	0.4
aa244-252	-0.8	-	-0.4	0.0	0.6	-0.2	0.4
aa253-261	0.0	-	0.0	0.0	0.0	-0.1	0.0
aa253-262	0.0	-	0.0	0.0	0.0	0.0	0.0
aa262-277	-0.9	-	-0.4	-0.4	0.0	-0.1	0.0
aa263-277	-0.8	-	-0.4	-0.4	0.0	-0.1	0.0
aa266-277	-0.1	-	0.1	-0.1	-0.1	0.0	0.0
aa271-277	-0.2	-	-0.1	-0.1	0.0	0.0	0.0
	Reduction in H/DX normalized on trastuzumab Degly [%], average values of n=6$^{(+)}$, 12$^{(++)}$ or 18$^{(+++)}$ replicates						
aa185-193	0.0±0.0	0.3±1.0	1.5±1.4	0.1±0.8	-0.5±0.7	1.2±1.9	0.7±0.8
aa186-197	0.0±0.0	0.0±0.6	0.9±1.4	0.0±0.4	-0.2±0.6	1.4±2.1	0.3±0.3
aa235-240	0.0±0.0	33.9±1.9	17.9±1.3	24.5±1.8	39.6±0.5	37.0±2.5	37.9±0.5
aa235-241	0.0±0.0	38.2±1.7	19.0±1.4	27.1±1.8	44.3±0.7	41.2±3.7	43.3±0.7
aa241-252	0.0±0.0	26.2±1.9	12.5±0.7	27.1±3	48.7±3.3	18.2±4.3	41.4±0.7
aa242-251	0.0±0.0	24.5±2.8	8.0±1.7	24.6±3.6	45.9±2.0	16.5±2.2	39.3±0.6
aa242-252	0.0±0.0	27.7±0.9	14.4±0.9	29±1.8	50.0±1.6	21.5±3	45.6±0.8
aa243-252	0.0±0.0	27.1±1.4	13.8±0.6	27.6±2.0	50.9±1.5	19.9±3.2	45.9±0.8
aa244-252	0.0±0.0	27.3±2.3	14.5±1.5	27.6±2.0	48.2±1.3	22.2±2.6	44.7±0.8
aa253-261	0.0±0.0	1.4±2.7	-0.9±1.6	0.2±2.0	1.0±1.9	0.6±1.9	0.2±2.3
aa253-262	0.0±0.0	0.7±2.6	-2.0±2.6	0.0±2.4	2.5±3.5	-0.2±3.3	-0.2±0.8
aa262-277	0.0±0.0	19.9±0.7	10.6±0.7	11.1±1.0	20.7±0.5	19.9±2.1	19.9±0.3
aa263-277	0.0±0.0	17.2±0.6	9.1±0.6	9.5±1.2	17.6±0.6	17.9±2.8	17.4±0.5
aa266-277	0.0±0.0	0.5±1.4	1.5±2.9	-0.7±1.2	0.0±1.2	2.8±3.9	3.0±1.6
aa271-277	0.0±0.0	8.9±4.2	6.5±1.5	4.4±2.3	7.1±3.6	11.6±1.5	10.0±0.8

Table S2. Relative FcγR binding of trastuzumab glycan variants (normalized with trastuzumab starting material), as shown in *Figure 4*.

	Trastuzumab	Trastuzumab RM	Trastuzumab Degly	Trastuzumab G0F	Trastuzumab G2F	Trastuzumab ST3	Trastuzumab ST6
	Relative binding, normalized [%], n=3						
FcRn	100±0.7	133.2±1.2	106.6±2.7	94.9±2.3	106.5±1.0	79.8±0.9	127.4±0.9
FcγRIa	100±0.1	103.0±0.1	31.6±0.2	96.6±0.2	102.2±0.1	94.2±0.0	100.4±5.3
FcγRIIa (R131)	100±0.7	107.3±0.4	1.0±0.0	95.4±0.5	93.9±0.7	64.1±0.6	129.5±2.2
FcγRIIa (H131)	100±0.1	98.3±0.2	0.8±1.6	89.7±0.1	102.3±0.2	74.9±0.6	119.9±0.2
FcγRIIb/c	100±3.7	103.1±3.5	-0.1±0.0	95.9±4.0	100.0±3.1	87.0±2.6	161.5±3.7
FcγRIIIa (V158)	100±0.2	105.3±0.1	0.9±1.6	94.5±0.2	120.4±0.1	78.2±0.5	121.3±0.1
FcγRIIIa (F158)	100±0.1	102.8±0.2	0.6±4.2	88.4±0.2	123.7±0.2	61.9±0.6	149.9±0.1

Table S3. D uptake differences of trastuzumab G2S2F ST3 vs. ST6, as shown in *Figure 5*.

	Deuterium uptake difference of trastuzumab G2S2F ST3 vs. trastuzumab G2S2F ST6 [Da], n=3						
	0.5 min	1 min	10 min	30 min	1 h	3 h	48 h
aa185-197	-0.1	-0.1	0.0	-0.1	0.0	0.0	0.0
aa189-197	-0.1	-0.1	0.0	0.0	0.0	0.0	0.0
aa235-240	0.1	0.1	0.4	0.3	0.3	0.1	0.0
aa235-241	0.1	0.1	0.3	0.4	0.3	0.2	0.2
aa241-251	0.1	0.1	0.6	0.6	0.5	0.3	0.1
aa242-251	0.1	0.2	0.6	0.6	0.5	0.3	0.0
aa242-252	0.1	0.1	0.6	0.6	0.6	0.3	0.0
aa243-252	0.1	0.1	0.5	0.5	0.5	0.3	0.1
aa244-251	0.1	0.1	0.5	0.5	0.4	0.2	0.0
aa244-252	0.1	0.1	0.6	0.6	0.5	0.3	0.0
aa245-252	0.1	0.1	0.5	0.5	0.4	0.3	0.0
aa253-261	-0.1	-0.1	0.1	0.1	0.1	0.3	0.1
aa253-262	-0.1	-0.1	0.0	0.0	0.0	0.1	0.0
aa257-274	0.0	-0.1	-0.1	0.0	0.0	0.0	0.0
aa262-277	0.1	0.1	0.2	0.3	0.4	0.3	0.0
aa263-275	0.1	0.1	0.4	0.6	0.7	0.6	0.0
aa263-277	0.1	0.1	0.3	0.5	0.6	0.5	0.0
aa266-275	0.0	0.0	0.0	0.0	0.0	0.1	0.0
aa266-277	0.0	0.0	0.0	0.0	0.0	0.0	0.0
aa270-277	0.0	0.0	0.0	0.0	0.0	0.1	0.0
aa271-277	0.0	0.0	0.0	0.1	0.1	0.1	0.0
aa278-285	0.0	0.0	-0.1	-0.1	0.0	0.1	0.0
aa278-300	1.7	2.4	1.2	-0.2	0.0	0.0	0.0
aa278-305	1.7	2.6	1.6	0.9	1.0	1.0	1.3
aa281-300	1.9	2.4	1.3	-0.1	-0.1	0.1	0.2
aa282-300	1.9	2.5	1.2	0.0	-0.1	-0.1	-0.1
aa282-306	1.8	2.4	1.6	0.8	0.8	1.0	1.4
aa282-313	0.1	0.1	0.1	0.4	0.4	0.6	0.2
aa297-325	1.7	2.3	1.6	0.7	0.8	1.0	1.3
aa301-306	0.0	0.0	0.0	0.0	0.1	0.2	0.5
aa307-315	0.0	0.0	0.0	0.0	0.1	0.1	0.0
aa307-318	0.0	0.0	0.0	0.0	0.0	0.1	0.1
